# Improvement of the quality of maize grain silage by a synergistic action of selected lactobacilli strains

**DOI:** 10.1007/s11274-017-2400-9

**Published:** 2017-12-18

**Authors:** K. J. Zielińska, A. U. Fabiszewska

**Affiliations:** 1 Department of Fermentation Technology, Prof. Wacław Dąbrowski Institute of Agricultural and Food Biotechnology, Rakowiecka 36 Street, 02-532 Warsaw, Poland; 2 Department of Chemistry, Faculty of Food Sciences, Warsaw University of Life Sciences, 159 c Nowoursynowska Street, 02-787 Warsaw, Poland

**Keywords:** Bacterial inoculants, Lactic acid bacteria, *Lactobacillus*, Silage quality

## Abstract

As silage is one of the most important feed sources for dairy cattle it is recommended for farmers to preserve silage by fermentation. Interaction of the five strains of *Lactobacillus* genera [*Lactobacillus buchneri* A KKP 2047 p (LB), *L. reuteri* M KKP 2048 p (LR), *L. plantarum* K KKP 593 p (LPk), *L. plantarum* S KKP 2021 p (LPs), *L. fermentum* N KKP 2020 p (LF)] has been shown aiming to increase the safety of corn grain silage fodder. Experiments were conducted in polyethylene microsilos for 48 days and on production scale in an experimental farm for 3 years. Synergistic activity of the studied bacterial strains in terms of reducing aflatoxin B_1_ and ochratoxin A levels was clear in these experimental variants wherein to the inoculants of the LB + LR strains subsequent bacterial strains LPk, LPs and LF were sequentially added. Silages inoculated with five bacterial strains were free from pathogens and showed the lowest yeast and mold count values among all experimental variants. As a result of employing the preparation starter culture for ensiling corn grain there were obtained silages characterized by high stability, microbiological and chemical purity, thus safe in feeding livestock.

## Introduction

Grain maize (*Zea mays* L.) is a key crop widely cultivated for animal feeding (Amon et al. 2007). Maize grain contains a lot of easily digestible carbohydrates and is low in fibers, which means that it constitutes a high energy forage for ruminant livestock. At the time of harvest maize contains 30–35% of water and this high humidity may often contribute to high contamination with aerobic bacteria, molds and mycotoxins. Immediately after threshing it should be preserved by drying, ensiling or by chemical preservation. As silage is one of the most important feed source for dairy cattle it is recommended for farmers to preserve silage by fermentation or by using chemical additives application due to lower costs in comparison to drying (Oude Elferink et al. 2001; Podkówka 2005; Richard et al. 2009).

The ensiling process is a complex one and involves interactions of numerous different chemical and microbiological processes. Several genera described as lactic acid bacteria (LAB) play a fundamental role in the ensiling process. LAB that are regularly associated with silages are members of the genera *Lactobacillus, Pediococcus, Leuconostoc, Enterococcus, Lactococcus* and *Streptococcus* (Lahtinen et al. 2012). Microbes of this group compete for available nutrients with microorganisms undesirable in the ensiling process, in particular clostridia, enterobacteria, bacilli, yeast and fungi. In difference with detrimental flora LAB have a positive effect on the quality of ensiled material because of their end-products of metabolism and the way of degradation of valuable nutrients (Muck and Pitt 1993; Lahtinen et al. 2012). Increased levels of undissociated volatile fatty acids, such as acetate, which are produced by LAB strains, may inhibit other microbes that initiate aerobic deterioration, especially rapid growth of yeast and molds, which causes silages to heat and spoil, decreasing its nutritional value. There is an evidence that some LAB produce antimicrobial and antifungal compounds and therefore are capable of inhibiting a considerable spectrum of bacteria and fungi. As a result of the synergistically acting strains of the homofermentative and heterofermentative LAB species of the genus *Lactobacillus* silages with increased microbiological quality may be obtained (Chan et al. 2008; Lindsey and Kung 2010; Muck 2013).

However, under farm conditions the populations of epiphytic LAB are not always large enough or do not have a composition suitable for promoting efficient homolactic fermentation (Davies et al. 2000). This is because the concept of LAB usage in silage inoculants has gained a favor in the last two decades especially with respect to *Lactobacillus* genera. The popularity is growing for reasons of health and safety, as well as nutritional quality (Davies et al. 2000; Zielińska et al. 2015a). Fermentation optimization is a field of study that has preoccupied many microbiologists. Often approach used to design silage additives is intuitive, seldom completely rational and based on past experience and knowledge, not taking into account many interactions between the optimized factors, which determine the outcome of the fermentation. Majority of bacterial additives stimulates uncontrolled fermentation of fodder (Muck 2013). What is important, it seems that strains added to the ensiled fodder could not be the same strains at later times (Parvin and Nishino 2010). On the basis of this facts it can be noted that strain selection could not be provided in laboratory flasks cultures, but a descriptive characteristics of tested microorganisms is still necessary. The alternative should be experiments in silos with an addition of a combination of well-known bacterial strains followed by final tests in farm conditions. This concept was used in this paper. The objective of this study was to investigate the synergistic action of LAB strains of the genus *Lactobacillus* applied as a starter culture in the ensiling process of maize grain. Addition of the bacteria to the ensiled plant material was supposed to improve the quality of silages, including microbiological quality, aerobic stability and a significant reduction of mycotoxin contamination, especially aflatoxin B_1_ and ochratoxin A.

## Materials and methods

### Bacterial strains

Five bacterial strains were examined in the study: *Lactobacillus buchneri* A KKP 2047 p, *L. reuteri* M KKP 2048 p, *L. plantarum* K KKP 593 p, *L. plantarum* S KKP 2021 p, *L. fermentum* N KKP 2020 p. Strains are deposited at the Culture Collection of Industrial Microorganisms at the Institute Agricultural and Food Biotechnology (IAFB) in Warsaw (Poland). Characteristics of lactobacilli strains was presented in Table 1.

**Table 1:** Characteristics of lactobacilli strains used in the study

Strain	Abilities	References
*L. buchneri* A KKP 2047 p	1,2-Propanediol synthesis and its metabolizing through 1-propanol to propionic acid	Zielińska et al. (2016)
*L. fermentum* N KKP 2020	Effectively decreasing aflatoxin B_1_ content and inhibiting *Aspergillus flavus* growth	Zielińska et al. (2012)
*L. plantarum* K KKP 593 p	Decreases mycotoxin levels and poses antagonistic activity against aerobic microorganisms	Zielińska et al. (2015a, b)
*L. plantarum* S KKP 2021 p	Decreases ochratoxin A content and poses antagonistic activity against aerobic microorganisms, particularly moulds and bacteria of the *Salmonella* genus and the *Escherichia* coli species	Zielińska et al. (2014)
*L. reuteri* M KKP 2048 p	Synthesis of cobalamin—a coenzyme for diol dehydratase catalyzing the step of propionic acid synthesis from 1,2-propanediol	Sriramulu et al. (2008)

### Bacterial inoculants

The bacterial inoculants contained LAB, carriers and emulsifiers according to the formula described previously (Zielińska et al. 2015a). Bacterial inoculants were produced in Prof. Wacław Dąbrowski Institute of Agriculture and Food Biotechnology in Warsaw, according to the manufacturing process described by Miecznikowski et al. (2008).

### Ensiling in microsilos

In experiments conducted in microsilos, relating to the synergistic activity of five bacterial strains in ensiled maize grain, the following experimental preparations were employed: *L. buchneri* A KKP 2047 p (LB), *L. reuteri* M KKP 2048 p (LR), *L. plantarum* K KKP 593 p (LPk), *L. plantarum* S KKP 2021 p (LPs), *L. fermentum* N KKP 2020 p (LF), *L. buchneri* A KKP 2047 p + *L. reuteri* M KKP 2048 p (LR + LB), *L. buchneri* A KKP 2047 p + *L. reuteri* M KKP 2048 p + *L. plantarum* K KKP 593 p (LB + LR + LPk), *L. buchneri* A KKP 2047 p + *L. reuteri* M KKP 2048 p + *L. plantarum* K KKP 593 p + *L. plantarum* S KKP 2021 p (LB + LR + LPk + LPs), *L. buchneri* A KKP 2047 p + *L. reuteri* M KKP 2048 p + *L. plantarum* K KKP 593 p + *L. plantarum* S KKP 2021 p + *L. fermentum* N KKP 2020 p (LB + LR + LPk + LPs + LF).

Maize grain was kept in polyethylene microsilos of 0.01 m^3^ capacity, sealed with a rubber stopper enabling release of gaseous products. Corn was treated with bacterial inoculants. A negative control without adding any starter culture was also prepared. All experiments were conducted using bacterial inoculants in a dose of 10 g t^−1^ of maize grain before ensiling. Silos were maintained at a temperature of 20 ± 2 °C for 48 days. Once a silo was opened, it was removed from the subsequent experiments. Experiments were made in triplicate, which meant that three silos were prepared for each treatment.

### The scheme of production scale experiments

Studies relating to the synergistic activity of bacterial strains were additionally conducted on production scale in an experimental farm. Maize grain silages having water content of around 40% were prepared in production silos. The scheme for production experiments was as follows: negative control, no inoculants added; positive control inoculated with a starter culture of the *L. buchneri* A KKP 2047 p (LB); experimental silage inoculated with a starter culture composed of five bacterial strains (LB + LR + LPk + LPs + LF). Bacterial inoculants were used in doses of 10 g t^−1^ of maize grain. Silages were maintained in 500 kg silos for 3 years, whereupon the silos were opened and three samples were collected from each silo, forming a representative sample when combined.

### Analytical procedures

The extract of macerated silages was prepared with distilled water, filtered through two layers of cheesecloth and used for chemical analyses. In all samples for control and experimental silages dry mass, pH, aerobic stability, the content of lactic, acetic, butyric and propionic acids, the content of 1,2-propanediol and 1-propanol and the content of aflatoxin B_1_ and ochratoxin A were determined according to the methods described previously (Zielińska et al. 2015a, b). 10 g of each silage was homogenised in 100 mL of sterile peptone water, mixed vigorously for 60 min and used for culturing. The methods for determination of LAB count, mold count, *Salmonella* sp. and *E. coli* counts were described by Zielińska et al. (2015a).

### Statistical analysis

Statistical analyses of the results were performed by repeated measurements with one-way ANOVA in STATISTICA 12.0 (Statsoft, Poland), followed by Tukey’s multiple comparison test. ***p***-values of *p* ≤ 0.05 were considered to be statistically significant. The Shapiro–Wilk test was used to determine whether the population was normally distributed. Brown–Forsythe test was used to assess the equality of variances for a variable calculated for groups.

## Results

### Effect of action of bacterial strains on maize grain silages ensiled in microsilos

**Fig. 1:**
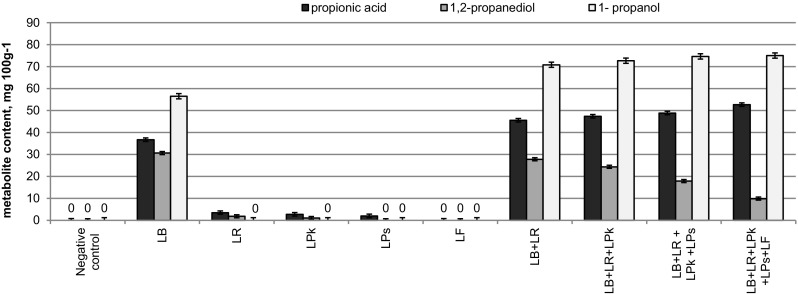
Propionic acid, 1,2-propanediol and 1-propanol content in maize grain silage treated with various bacteria after 48 days of ensiling

Individual bacterial strains comprised in the full preparation starter culture were characterized by special biochemical traits, specific for the particular strains (Table 1). The results of the experiments, relating to the effect of individual bacterial strains and synergistic activity thereof in the process of maize grain ensiling for 48 days in microsilos were shown in Table 2 and in Fig. 1.

**Table 2:** Chemical and microbial composition of maize grain silages treated with various bacterial inoculants after 48 days of ensiling in microsilos

	Negative control	LB	LR	LPk	LPs	LF	LB + LR	LB + LR + LPk	LB + LR + LPk + LPs	LB + LR + LPk + LPs + LF	SD
DM (%)	57.2^a^	60.8^c^	59.8^b^	59.5^b^	59.9^b^	60.2^bc^	60.9^c^	60.8^c^	60.2^bc^	60.9^c^	0.3
pH	4.82^a^	4.12^b^	4.18^b^	4.08^bc^	4.08^bc^	4.00^c^	4.08^bc^	3.96^c^	3.94^c^	3.90^c^	0.13
Lactic acid (g kg^−1^)	11.8^a^	17.2^b^	16.8^b^	18.4^bc^	18.2^b^	18.0^b^	17.8^b^	18.8^bc^	18.2^b^	19.6^c^	2.0
Acetic acid (g kg^−1^)	3.8^a^	4.7^b^	3.7^a^	4.6^b^	4.1^a^	3.9^a^	5.1^b^	4.8^b^	4.7^b^	4.8^b^	0.6
Propionic acid (mg 100 g^−1^)	0^a^	36.72^c^	3.5^b^	2.72^b^	2.00^b^	0^a^	45.54^d^	47.36^d^	48.84^d^	52.68^d^	0.8
1,2-Propanediol (mg 100 g^− 1^)	0^a^	30.64 ^g^	1.83^b^	1.04^b^	0^a^	0^a^	27.80^f^	24.36^e^	17.89^d^	9.86^c^	0.7
1- propanol (mg 100 g^−1^)	0^a^	56.52^b^	0^a^	0^a^	0^a^	0^a^	70.84^c^	72.67^c^	74.65^c^	75.05^c^	1.2
LAB (log cfu g^−1^)	7.30^a^	8.02^ab^	7.60^a^	8.60^b^	8.30^b^	8.00^ab^	8.00^ab^	8.80^b^	8.70^b^	8.80^b^	0.50
Yeast (log cfu g^−1^ DM^−1^)	4.60^c^	2.90^a^	3.30^b^	2.50^a^	2.60^a^	2.90^a^	2.60^a^	2.30^a^	> 2.00	> 2.00	0.60
Molds (log cfu g DM^−1^)	6.30^a^	4.00^b^	4.30^b^	3.60^bc^	3.30^c^	4.00^b^	3.30^c^	2.30^d^	2.00^d^	2.00^d^	0.70
*Salmonella* sp. (log cfu g DM^−1^)	2.60^a^	0^b^	> 2.00	0^b^	0^b^	> 2.00	0^b^	0^b^	0^b^	0^b^	0.20
*E. coli* (log cfu g DM^−1^)	3.60^a^	2.00^b^	2.00^b^	0^c^	0^c^	2.00^b^	0^c^	0^c^	0^c^	0^c^	0.20
Coliform (log cfu g DM^−1^)	3.90^d^	2.30^c^	2.60^c^	1.00^b^	2.00^bc^	2.30^c^	2.00^bc^	1.00^b^	1.00^b^	0^a^	0.50
Alfatoxin B_1_ in DM (ppb)	13.60^f^	10.50^e^	12.98^f^	10.50^e^	9.85^d^	4.62^b^	9.00^d^	7.40^c^	4.56^b^	2.75^a^	0.32
Ochratoxin A in DM (ppb	16.30^a^	12.96^b^	15.38^a^	8.06^d^	4.34^f^	7.90^d^	10.38^c^	5.87^e^	3.78^f^	3.30^f^	0.38
Aerobic stability (h)	72^a^	162^b^	158^b^	172^b^	170^b^	156^b^	180^bc^	205^c^	216^c^	218^c^	21

Statistically different groups marked with different letters according to Tukey’s test

Irrespectively the kind of bacterial inoculant used in ensiling of maize grain in microsilos a significant pH decrease from 3.90 to 4.18 was observed. In turn, particular silages differed in metabolite profiles depending on starter culture formulation. The highest content of lactic acid was detected in silages prepared with the addition of all five strains and the lowest in the LR silage (16.8 g kg^−1^). In this variant, the lowest LAB cells count (< 8 cfu g DM^−1^) was determined, which may indicate that the strain had the lowest capability to grow in maize grain environment. As predicted by the individual characteristics of the tested strains, the highest acetic acid content was observed in silage prepared with *L. buchneri* KKP 2047 p (5.1 for LB + LR and 4.7 g kg^−1^ for LB). The emergence of propionic acid and 1-propanol in silage after 48 days of microsilos experiments was also directly related to the presence of *L. buchneri* in the starter culture (36.72 and 56.52 mg 100 g^−1^ respectively) and the content of these metabolites increased when the formulation was enriched with *L. reuterii* (45.54–52.68 and 70.84–75.05 mg 100 g^−1^ respectively).

Synergistic activity of the studied bacterial strains in terms of reducing aflatoxin B_1_ and ochratoxin A levels is clear in these experimental variants wherein to the inoculants of the LB + LR strains subsequent bacterial strains LPk, LPs and LF were sequentially added. Due to joint activity of these bacterial strains aflatoxin B_1_ and ochratoxin A levels were decreased by about 80% relative to control silage and by about 74% relative to silage inoculated with the LB strain only. An interaction of the bacterial strains in terms of reducing yeast and mold counts and potentially pathogenic bacteria was also shown. Silages inoculated with five bacterial strains were free from pathogens and showed the lowest yeast and mold count values among all experimental variants.

### Long-term experiment in farm conditions

**Fig. 2:**
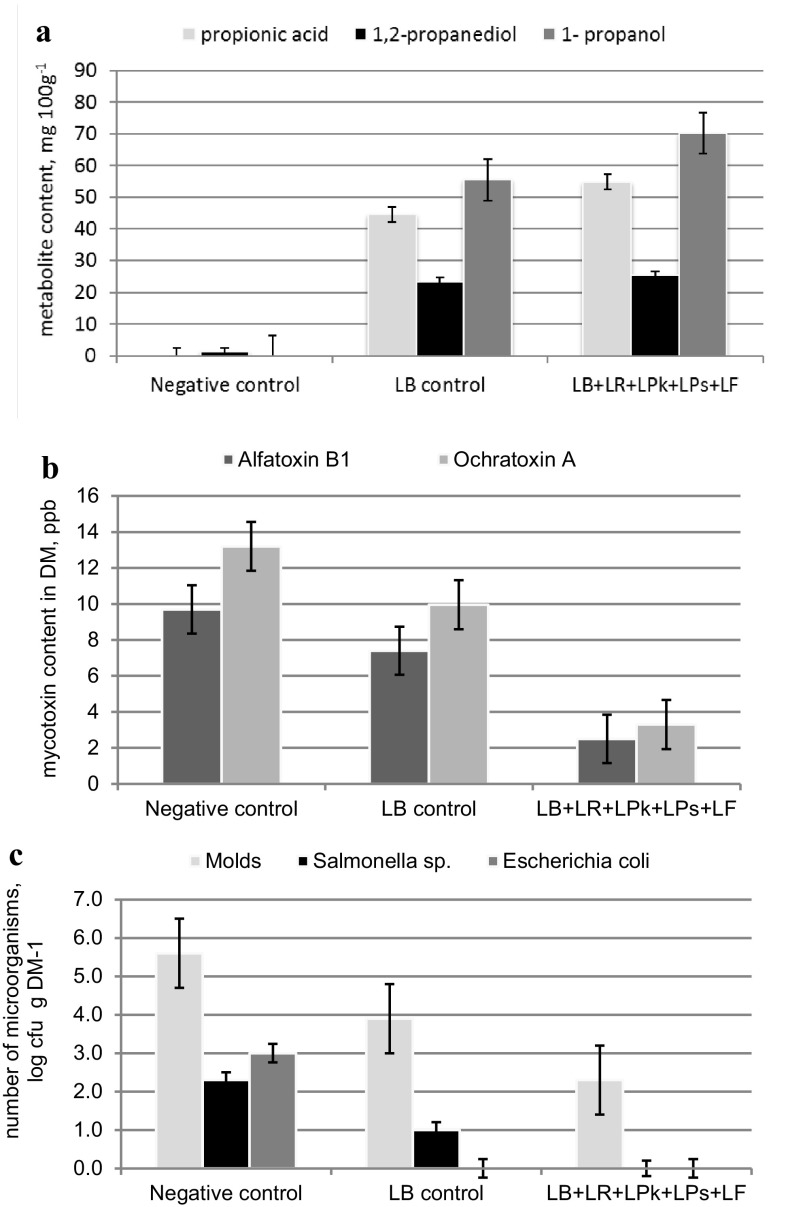
Comparison of selected lactobacilli strains action in maize grain silages after 3 years of silage preservation under farm conditions: **a** 1,2-propanediol and its metabolites content; **b** mycotoxin contamination; **c** number of microorganisms affecting microbiological quality of silage

The results of production scale studies relating to the effects of interaction of the five bacterial strains relative to the activity of the *L. buchneri* A KKP 2047 p strain added alone to the maize grain are shown in Table 3 and in Fig. 2a–c. Silages used as negative controls were characterized by high pH—5.66, low lactic acid level—3.2 g kg^−1^, high butyric acid level—1.25 g kg^−1^, no propionic acid and 1-propanol, trace levels of 1,2-propanediol, aerobic stability of 4 days, and were furthermore contaminated with molds, pathogenic bacteria and aflatoxin B_1_ and ochratoxin A. The studied parameters disqualified the control silages for use as animal fodder. Silages inoculated with *L. buchneri* A KKP 2047 p (LB) strain culture were characterized by pH—4.38, lactic acid level—14.8 g kg^−1^ with no butyric acid. These silages were determined to contain significant amounts of 1,2-propanediol and products of conversion thereof: 1-propanol and propionic acid, 44.59, 23.36, 55.48 mg 100 g^−1^ respectively, lower mold count, and aflatoxin B_1_ and ochratoxin A levels reduced by about 24–25% relatively to negative control.

**Table 3:** Chemical and microbial composition of maize grain silage treated with various bacteria after 3 years of silage preservation under farm conditions

	Negative control^*^	LB control	LB + LR + LPk + LPs + LF^1^	SD
DM (%)	60.0^a^	62.7^b^	63.2^b^	0.15
pH	5.66^a^	4.38^b^	4.06^c^	0.03
Lactic acid (g kg^−1^)	3.2^a^	14.8^b^	17.3^b^	2.1
Acetic acid (g kg^−1^)	4.9^a^	4.2^a^	3.9^a^	0.18
Butyric acid (g kg^−1^)	1.25^b^	0^a^	0^a^	0.12
Propionic acid (mg 100 g^−1^)	0^a^	44.59^b^	54.80^c^	2.4
1,2-Propanediol (mg 100 g^−1^)	1.20^a^	23.36^b^	25.47^b^	1.2
1-Propanol (mg 100 g^−1^)	0^a^	55.48^b^	70.33^c^	6.5
LAB (log cfu g DM^−1^)	5.30^a^	6.30^b^	6.92^b^	0.37
Molds (log cfu g DM^−1^)	5.60^c^	3.90^b^	2.30^a^	0.90
*Salmonella* sp. (log cfu g DM^−1^)	2.30^c^	1.00^b^	0^a^	0.20
*E. coli*. (log cfu g DM^−1^)	3.00^b^	0^a^	0^a^	0.24
Aflatoxin B1 in DM (ppb)	9.70^c^	7.40^b^	2.50^a^	1.34
Ochratoxin A in DM (ppb)	13.20^c^	9.96^b^	3.30^a^	1.36
Aerobic stability (days)	4^a^	12^b^	16^c^	1.5

Statistically different groups marked with different letters according to Tukey’s test

^1^The results as shown in the patent application (Zielińska et al. 2016)

In the production scale experiment the synergistic activity of the five bacterial strains of the *Lactobacillus* genus comprised in the preparation was confirmed in comparison to negative control and control inoculated with *L. buchneri* A KKP 2047 p (LB) strain culture in terms of the following. 1,2-propanediol synthesis and its metabolism changed among silages, an increase in the levels of the intermediate compound, 1-propanol (up to 70.33 mg 100 g^−1^ of silage) and the final product—propionic acid (up to 54.80 mg 100 g^−1^ silage) was observed. Decrease in mold count to 2.30 log cfu g DM^−1^ and elimination of potentially pathogenic bacteria was noted. Decrease in aflatoxin B_1_ and ochratoxin A levels in silages by 74 and 75% respectively relative to negative control and by 65 and 67% respectively relative to LB control were a fact. Unfortunately, an increase in aerobic stability of silages by 12 days relative to negative control and by 4 days relative to LB control.

## Discussion

Microbial inoculants have become the dominant silage additive type in most parts of the world and most of these products are strains of facultative heterofermentative and heterofermentative LAB (Muck 2013). It has been investigated in the paper whether the use of selected lactobacilli strains in silage inoculants could improve the quality of maize grain silage. Five strains of *Lactobacillus* genus were examined in many combinations in order to evaluate its synergistic action. The issues to be evaluated were as follows: improvement in aerobic stability, advantageous metabolic profile of silages as well as improvement in microbiological and mycotoxin contamination decrease. Based on the obtained results for studies conducted on laboratory and production scale, synergistic activity of the selected strains derived from four bacterial species of the *Lactobacillus* genus, has been demonstrated, being due to their characteristic biochemical traits.

The use of starter cultures ensure that there is an immediate decrease in the pH to prevent growth of undesirable microorganisms and so-called aerobic deterioration. The aerobic stability of silages is enhanced also by many low mass organic acids, such as acetic acid, lactic acid and propionic acid and some other metabolites (e.g. 1,2-propanediol), which occurrence in silages is associated with LAB growth (Danner et al. 2003). It has been demonstrated that *L. buchneri* has a metabolic pathway to degrade lactic acid anaerobically and converse it to acetic acid, 1,2-propanediol and traces of ethanol (Oude Elferink et al. 2001). Nishino et al. (2003) concluded that inoculation of *L. buchneri* to whole crop maize causes accumulation of 1,2-propanediol in silages without further degradation into propionic acid and 1-propanol. Krooneman et al. (2002) isolated and identified new lactobacilli species *L. diolivorans* able to use 1,2-propanediol as a sole carbon source under anoxic conditions and to convert the metabolite to propionate. The *L. diolivorans* LMG 19,667(T) strain of Charley and Kung (2005), most probably only has the ability to metabolize this compound when it is already present in the environment as previously shown in literature (Krooneman et al. 2002). Thus, in view of the data (Zielińska et al. 2016) one of the synergistically interacting five bacterial strains used in the study, *L. buchneri* A KKP 2047 p additionally has new and unique for the species features of both synthesizing and metabolizing 1,2-propanediol to 1-propanol and propionic acid. The strain is not solely responsible for propionic acid occurrence in the maize grain silage. There should be mentioned the role of *L. reuteri* in formation of this compund. Capacity of synthesizing cobalamin-dependent diol dehydratase allows *L. reuteri* to carry out a disproportionation reaction converting 1,2-propanediol to propionate and propanol (Sriramulu et al. 2008). Biological additives based on the propionate-producing propionibacteria appear to be less suitable for the improvement of silage aerobic stability, due to the fact that these bacteria are only able to proliferate and produce propionate if the silage pH remains relatively high (Weinberg and Muck 1996).

Synergistic activity of bacterial strains, in terms of increase in 1,2-propanediol, 1-propanol and propionic acid levels and in terms of enhancing aerobic stability, is visible for all experimental variants for co-fermentation with the studied strains, and in particular in silages inoculated with a starter culture of the following strains: *L. buchneri* A KKP 2047 p (LB) and *L. reuteri* M KKP 2048 p (LR). Volatile fatty acids such as propionic acid and acetic acid are much better inhibitors of yeasts than is lactic acid, and that mixtures of lactic acid and propionic or acetic acid have a synergistic inhibitory effect (Moon 1983). Indeed, the presence of these metabolites had a positive result on increase of aerobic stability. For LB + LR + LPk preparation the resistance to aerobic deterioration by spoilage microorganisms was high (> 200 h) and it did not change significantly after addition *L. plantarum* S and *L. fermentum* to the starter culture (215 and 218 h respectively).

Not to mention the aspects of antimicrobial abilities of LAB against pathogens, molds and yeast. For the improvement of feed safety, the aspect of the ability of mycotoxins detoxification by LAB strains, including aflatoxin B_1_ and ochratoxin A are important. The ability to remove these toxins from the environment through adsorption and/or enzymatic degradation is characteristic only for the few bacterial strains of the genus *Lactobacillus* (Fuchs et al. 2008), hence the starter culture composition for plant preservation is indicated as it was shown in this study. Yeasts are generally the initiators of aerobic deterioration, consuming sugars and fermentation acids and arising pH and silage temperature (Muck 2013). The PCT application by Colombatti et al. (2014) revealed a synergistic composition comprising a mix of bacteria of the genera *Lactobacillus* and *Propionibacterium* which was particularly useful to reduce or eliminate contamination by bacteria of the genus *Salmonella* and fungi, thus also preventing the occurrence of mycotoxins in soybean meal.

As a result of employing the preparation starter culture, having the five bacterial strain composition, maize grain silages were obtained having high stability, microbiological and chemical purity, thus completely safe in feeding livestock even after 3 years of preservation. There is not many similar studies performed during ensiling of fodder to the authors’ knowledge. Only few reports deal with the synergistic effect of individual lactobacilli strains in starter cultures on silage quality. Zhang et al. (2009) investigated the effect of *L. buchneri* alone or in combination with *L. plantarum* on the fermentation, aerobic stability, bacteria diversity and ruminal degradability of alfalfa silage. The *L. buchneri* and *L. plantarum*-inoculated silage had more acetic acid and less yeast as well as lower NH_3_–N/TN than control. Inoculating LAB inhibited harmful microorganisms, such as *Enterobacterium* and *Klebsiella pneumonia* and improved aerobic stability of fodder (Zhang et al. 2009). Similar studies provided Comino et al. (2014) inoculating maize from harvest I, I, III and IV with a commercial inoculant containing *L. casei* and *L. buchneri*. And once again, inoculation lowered yeast and mold counts, increase 1,2-propanediol and acetic acid, pH, DM losses and aerobic stability (to over 200 h), but for a change, no differences were observed between the treated and untreated silages harvested at the last stage of maturity. Noteworthy, it cannot be summarized that the inoculation in that particular experiments enhanced milk production (Comino et al. 2014). Some recent experiments aimed to investigate the effect of LAB silage inoculants on dry matter intake, digestibility, milk yield, milk composition, and methane (CH_4_) production from dairy cows in vivo. Results of this study indicated minimal responses in animal performance to inoculation of grass silage with mixture of *L. plantarum, L. lactis* and *L. buchneri*. Dose differences as well as different basal silages and ensiling conditions were likely responsible for the lack of significant effects observed here (Ellis et al. 2016).

The approach that one takes in selecting strains depends upon the goals (Muck 2013). It seems that the most difficult target today is improvement in animal performance and there is still a lot to do to analyze for the minor fermentation products not only on laboratory scale but also in the field conditions.

## References

[CR1] Amon T, Amon B, Kryvoruchko V, Zollitsch W, Mayer K, Gruber L (2007). Biogas production from maize and dairy cattle manure—influence of biomass composition on the methane yield. Agric Ecosyst Environ.

[CR2] Chan RK, Dennis SM, Harman EK, Hendrick CA, Ruser BG, Rutherford V, Smiley BK, Wortman C (2008) *Lactobacillus buchneri* strain LN5689 and its use to improve aerobic stability of silage. Patent USA no. US 2008/0138463

[CR3] Charley R, Kung JR (2005) Treatment of silage with *Lactobacillus diolivorans*. Patent USA no. US 2005/0281917 A1

[CR4] Colombatti F, Palacios LE, Ventrici EO (2014) A synergistic composition comprising a mix of bacteria of the genera *Lactobacillus* and *Propionibacterium freudenreichii* ssp. *shermani* and uses thereof. PCT Patent Application WO2015161877 A1

[CR5] Comino L, Tabaco E, Righi F, Revello-Chion A, Quarantelli A, Borreani G (2014). Effects of an inoculant containing a *Lactobacillus buchneri* that produces ferulate-esterase on fermentation products, aerobic stability, and fibre digestability of maize silage harvested at different stages of maturity. Anim Feed Sci Technol.

[CR6] Danner H, Holzer M, Mayrhuber E, Braun R (2003). Acetic acid increases stability of silage under aerobic conditions. Appl Environ Microbiol.

[CR7] Davies ZS, Gilbert RJ, Merry RJ, Kell DB, Theodorou MK, Griffith GW (2000). Efficient improvement of silage additives by using genetic algorithms. Appl Environ Microbiol.

[CR8] Ellis JL, Hindrichsen IK, Klop G, Kinley RD, Milora N, Bannink A, Dijkstra J (2016). Effects of lactic acid bacteria silage inoculation on methane emission and productivity of Holstein Friesian dairy cattle. J Dairy Sci.

[CR9] Fuchs S, Sontag G, Stidl R, Ehrlich V, Kundi M, Knasmüller S (2008). Detoxification of patulin and ochratoxin A, two abundant mycotoxins, by lactic acid bacteria. Food Chem Toxicol.

[CR10] Krooneman J, Faber F, Alderkamp AC, Qude Elferink SJHW, Driehuis F, Cleenwerck I, Swings J, Gottschal JC, Vancanneyt M (2002). *Lactobacillus diolivorans* sp. nov. a 1,2-propanediol-degrading bacterium isolated from aerobically stable maize silage. Int J Syst Evol Microbiol.

[CR11] Lahtinen S, Ouwehand AC, Salminen S, von Wright A (2012). Lactic acid bacteria. Microbiological and functional aspects.

[CR100] Lindsey JR, Kung L (2010). Effects of combining *Lactobacillus buchneri* 40788 with various lactic acid bacteria on the fermentation and aerobic stability of corn silage. Anim Feed Sci Tech.

[CR12] Miecznikowski A, Zielińska K, Suterska A, Stecka K (2008) Sposób wytwarzania granulowanego preparatu bakterii fermentacji mlekowej. Patent Poland no. PL 214654 (patent in Polish)

[CR101] Moon NJ (1983). Inhibition of the growth of acid tolerant yeasts by acetato, lactate and propionate and their synergistic mixtures. J Appl Bacteriol.

[CR13] Muck RE (2013). Recent advances in silage microbiology. Agric Food Sci.

[CR14] Muck RE, Pitt RE (1993) Ensiling and its effect on crop quality. In: Proceedings of the national silage production conference, Syracuse, New York, USA, pp 57–66

[CR15] Nishino N, Yoshida M, Shiota H, Sakaguchi E (2003). Accumulation of 1,2-propanediol and enhancement of aerobic stability in whole crop maize silage inoculated with *Lactobacillus buchneri*.. J Appl Microbiol.

[CR16] Oude Elferink SJWH, Krooneman J, Gottschal JC, Spoelstra SF, Faber F, Driehuis F (2001). Anaerobic conversion of lactic acid to acetic acid and 1,2-propanediol of *Lactobacillus buchneri*. Appl Environ Microbiol.

[CR17] Parvin S, Nishino N (2010). Succession of lactic acid bacteria in wilted rhodegrass silage assessed by plate culture and denaturing gradient gel electrophoresis. Grassland Sci.

[CR18] Podkówka Z (2005). Kukurydza w żywieniu zwierząt. Agroserwis.

[CR19] Richard E, Heutte N, Bouchart V, Garon D (2009). Evaluation of fungal contamination and mycotoxin production in maize silage. Anim Feed Sci Technol.

[CR20] Saksena RK, Anand P, Saran S, Isar J (2009). Microbial production of 1,3-propanediol: recent developments and emerging opportunities. Biotechnol Adv.

[CR21] Sriramulu DD, Liang M, Hernandez-Romero D, Raux-Deery E, Lunsdorf H, Parsons JB, Warren MJ, Prentice MB (2008). *Lactobacillus reuteri* DSM 20016 produces cobalamin-dependent diol dehydratase in metabolosomes and metabolizes 1,2-propanediol by disproportionation. J Bacteriol.

[CR22] Taranto MP, Vera JL, Hugenholtz J, de Valdez GF, Sesma F (2003). *Lactobacillus reuteri* CRL1098 produces cobalamin. J Bacteriol.

[CR23] Weinberg ZG, Muck RE (1996). New trends and opportunities in the development and use of inoculants for silage. FEMS Microbiol Rev.

[CR24] Zhang T, Li L, Wang X-f, Zeng Z-h, Hu Y-g, Cui Z-j (2009). Effects of *Lactobacillus buchneri* and *Lactobacillus plantarum* on fermentation, aerobic stability, bacteria diversity and ruminal degradability of alfalfa silage. World J Microbiol Biotechnol.

[CR25] Zielińska K, Suterska A, Miecznikowski A, Stecka K, Kupryś M (2012) Szczep bakterii *Lactobacillus fermentum* N. Patent Poland no. PL 211530 (patent in Polish)

[CR26] Zielińska K, Stecka K, Miecznikowski A, Kapturowska A, Kupryś M (2014) Strain *Lactobacillus plantarum* S, the use of the strain of *Lactobacillus plantarum* S and the preparation for roughages ensiling. Patent USA no. US 8.697.423

[CR27] Zielińska K, Fabiszewska A, Stefańska I (2015). Different aspects of *Lactobacillus* inoculants on the improvement of quality and safety of alfalfa silage. Chil J Agric Res.

[CR28] Zielińska K, Fabiszewska A, Wróbel B (2015). A contamination of sward from the grasslands of organic and conventional farms with aflatoxins and ochratoxin A. J Res Appl Agric Eng.

[CR29] Zielińska K, Fabiszewska A, Stecka K, Świątek M (2016) Strain *Lactobacillus buchneri* A, composition, a multi-component preparation for starch- rich plant preservation, their use and a method for plant preservation. Patent USA no. US 9.370.199

